# Supplementary material for: Structural basis for the activation of acid ceramidase
Source: Nat Commun. 2018 Apr 24;9:1621. doi: 10.1038/s41467-018-03844-2 (PMC5915598; doi:10.1038/s41467-018-03844-2)
Supplement: Supplementary file 2 — Supplementary Information [file 41467_2018_3844_MOESM2_ESM.pdf]

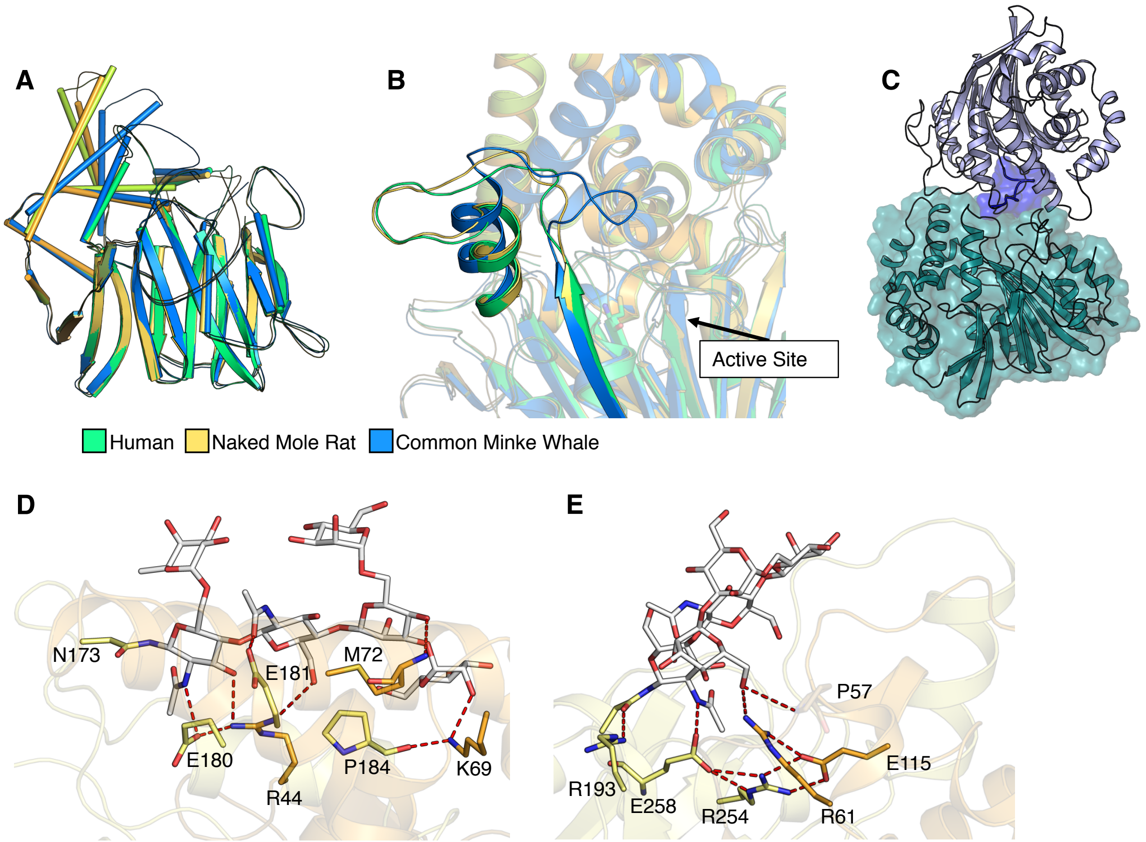

Supplementary Figure 1| Differences in structures and the network formed by glycosylation. (**A**) Comparison of the structures of the inactive aCDase from naked mole rat with the common minke whale (sequence identity=83%, r.m.s.d.=0.73 Å for 3589 corresponding atoms) and with the mature human-aCDase (sequence identity=83%, r.m.s.d.=0.61 Å for 4089 corresponding atoms). (**B**) Difference in the positioning of the loop connecting strand β4 to helix α6 (L4-6). In cmw-aCDase, L4-6 is oriented towards the active site, whereas in both human and nmr-aCDase, it is pointing towards the exterior. (**C**) In the cmw-aCDase conformation, L4-6 (dark blue surface) forms symmetry-related crystal contacts with the same region in another molecule. (**D, E**) Glycosylation of Asn 173 and Asn 259 leads to extensive bridging interactions between the two subunits. In **D**, the glycan of Asn 173 hydrogen bonds with Arg 44, Lys 69, and with the backbone of Met 72 of the α-subunit. Additionally, the glycan interacts with Glu 180 to properly position it for

hydrogen-bonding with Arg 44. In **E**, the glycan of Asn 259 hydrogen bonds with Pro 57 and Arg 61 of the α-subunit, and with three residues from the β-subunit (Arg 193, Glu 258, Arg 254). The glycans are shown as white sticks; residues of the α-subunit, as orange sticks, and residues of the β-subunit as yellow sticks.

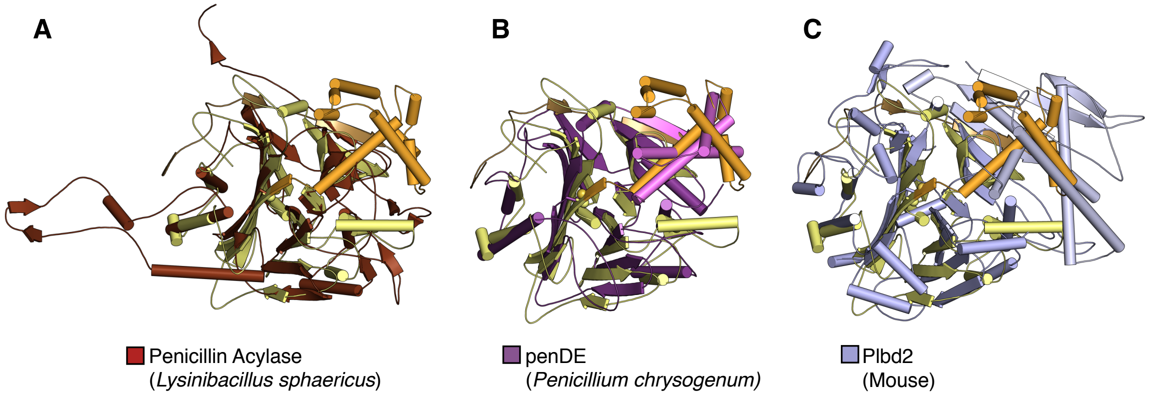

Supplementary Figure 2| Comparison of the structure of aCDase with structurally related Ntn-hydrolases. The structure of inactive aCDase is superimposed on other Ntn-hydrolases, such as (**A**) the Penicillin Acylase (PDB code 2PVA, overall sequence identity=19%, r.m.s.d.=0.88 Å for 243 corresponding α-carbon atoms) which lacks an α-subunit, (**B**) penDE (PDB code 2X1D, overall sequence identity=11%, r.m.s.d.= 3.01 Å for 1202 corresponding α-carbon atoms), and (**C**) Plbd2 (PDB code 3FBX, overall sequence identity=11%, r.m.s.d.= 2.09 Å for 62 corresponding α-carbon atoms). The α-subunit of nmr-aCDase is colored in orange and the β-subunit, in yellow. The active site is shown as an orange sphere for penicillin acylase, penDe, and Plbd2, and as a yellow sphere for aCDase.

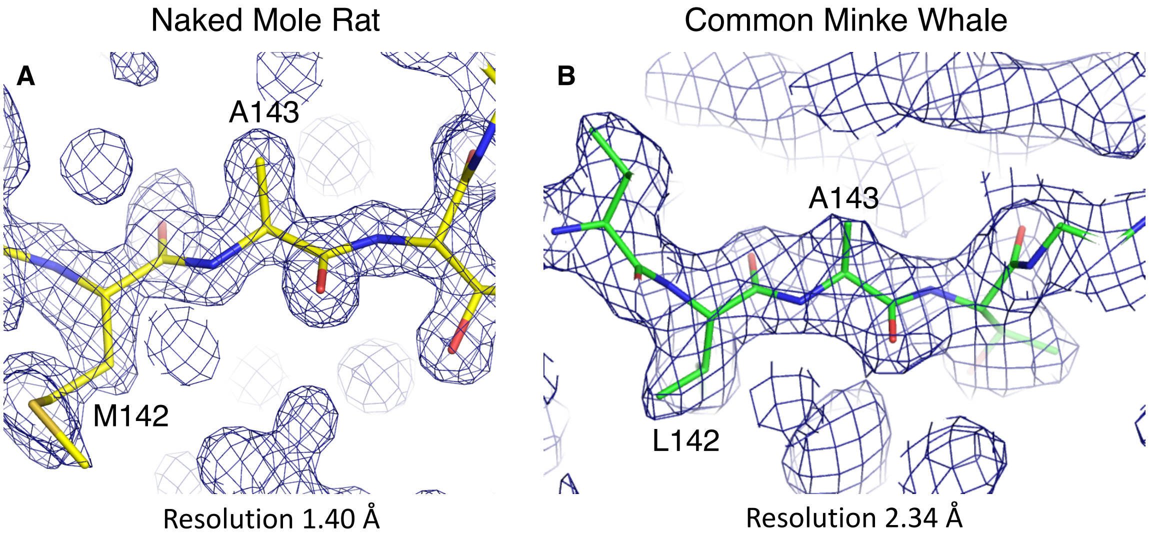

Supplementary Figure 3| Electron density of intact active site peptide bond in the proenzyme. (**A, B**) Final 2Fo-Fc electron density maps contoured at 1.2 sigma.

```
Human       MPGRSCVA--L---VLLAAAVSCAVAQHAPPWTEDCRKSTY-PPSGPTYRGAVPWYTINL 54
NMR         MLGRSRLT--F---VLLAAAVTCAEAQHAPPWTEDCRKSTY-PPSGPTYRGPVPWYTINL 54
CMW         MLGRSRLT--F---VLLSVTVTCSVAQHVPPWTEDCRKSTY-PPSGPTYRGPVPWYTINL 54
X.Laevis    -----MSALPLRLLLCFVVASLSGLAQDVPPYTEDCRSGTY-PPSGPTFKGNVSWYTVNL 54
Mouse       MRGQSLLT--W---V-LAAAVTCAQAQDVPPWTEDCRKSTY-PPSGPTYRGPVPWHTINL 53
C.Elegans   MLR------ELSV-LLLVAVCAAKHVELPAPFKDHCILDDKQNLYDPSKQFDIKWYDVNL 53
Bovine      MLGWSRLT--F---ILLSGIVTCLVAQQVPPWTEDCRKSTY-PPSGPTYRGPVPWYTINL 54
Cat         MLGWGRLT--F---VLLTVVATCAVAQNAPPWTEDCRKSTY-PPSGPTYRGPVPWYTINL 54
Chicken     MAGRGRALLPPAVLM-----VLVLLVVAPDPYGEDCRSKMY-PPSGPTFKGNVPTYIINL 54
                          :          .    *: :.*         .*: :  :  : :**

Human       DLPPYKRWHELMLDKAPVLKVIVNSLKNMINTFVPSGKIMQVVDEKLPGLLGNFPGPFEE 114
NMR         DLPPYKRWHELMVDKGPMLKIIVNSFKNMVNTFVPSGKVMQMVDQKLPDLLGQFSGPYEE 114
CMW         DLPPYKRWHELMVDKAPALKVIVNYLKNMINAFEPSGKIVQLVDQKLPGLLGSFPGPFEE 114
X.Laevis    DQAPQERWQKLISEKKTALSILIQAIKDLATSFFPSEKIIKLVDTKLPLLIGTLPCPFGE 114
Mouse       DLPPYKRWHELLAQKAPALRILVNSITSLVNTFVPSGKLMKMVDQKLPGMIGSLPDPFGE 113
C.Elegans   DLPPSERWVQIATANKEHIADLIGVLINLITPWFPNA--IDFVDDVFGDLAPKLAQPYRD 111
Bovine      DLPPYKRWHELMVVKAPALKVIVNSMKNIVNAFVPSGKIIHLVDQKLPGLLGNFPGPFEE 114
Cat         DLPPYKRWHELMADKAPALKVIMNSLKNMINAFVPSGKIIQIVDQKLPGLLGNFPGPYEE 114
Chicken     DLPPSKRWDELIRAKKTELKAVIQNIKDIANTFFPSGKIVDIVDHKISHLTDTLPYPFNE 114
            *  * :** ::   :   :  ::   : .: . : *.   :..**  :   :   :  *: :

Human       EMKGIAAVTDIPLGEIISFNIFYELFTICTSIVAEDKKGHLIHGRNMDFGVFLGWNINND 174
NMR         EMKGIADVTEIPLGEIISFNIFYELFTMCTSIITEDKKGHLLHVRNMDFGIFLGWNINNN 174
CMW         EMKGIAAVTEIPLGEIILFNIFYEFFTICTSIITEDKEGHLLHARNMDFGVFLGWNVNNN 174
X.Laevis    EIKGIADASGLPLGEVMLFNIFYEVFTVCTSVVAEDKSGKLYHARNLDFGLFLGWDVKNN 174
Mouse       EMRGIADVTGIPLGEIISFNIFYELFTMCTSIITEDEKGHLLHGRNMDFGIFLGWNINNN 173
C.Elegans   EIFSIANATGIPLGQITMYNIFYEIFTVCTSVIAQDKDGHVFHARNLDFGLFMGWDPVLH 171
Bovine      EMKGIAAVTEIPLGEIILFNIFYEFFTICTSIITEDKEGHLLHGRNLDFGVFLGWNINND 174
Cat         EMKGIAAVTDIPLGEIISFNVFYEIFTICTSVITEDKQGHLLHGRNLDFGIFLGWNINNN 174
Chicken     ELQGIANSSGIPLGEIVIFNIFYEIFTVCTSIVAEDSRGKLYHARNLDFGLFLGWDVKNN 174
            *: .**  : :***::  :*:***.**:***::::*. *::  * **:***:*:**:   .

Human       TWVITEQLKPLTVNLDFQRNNKTVFKASSFAGYVGMLTGFKPGLFSLTLNERFSINGGYL 234
NMR         TWVITEELKPLTVNLDFQRNSKTVFKATSFAGYVGMLTGFKPGQFSLTLNERFSMNGGYL 234
CMW         TWVVTEELKPLTVNLDFQRNSKTVFKAAGFAGYVGMLTGFKPGLFSLTLNERFSTNGGFM 234
X.Laevis    SWMVTQLLRPLVVNVDFQRNGKTVFVSTSFAGYVGMLTGMKPGIFSLTMNERFSIDGGYI 234
Mouse       TWVVTEELKPLTVNLDFQRNNKTVFKATSFVGYVGMLTGFKPGLFSLSLNERFSINGGYL 233
C.Elegans   DWQISQKLRKMIINVNWLKDGKLLYKSNNFAGYIGIYNGLKPNAFSLTADDRFQLVGGYY 231
Bovine      TWVITEELKPLTVNLDFQRNNKTLFKATTFAGYVGMLTGFKPGLFSVTLNERFSIDGGFM 234
Cat         TWVVTEQLKPLTVNLDFQRNNKTVFKASSFAGYVGMLTGFKPGLFSLTLNERFGVNGGYM 234
Chicken     SWTVTRELKPTVVNLDFQRNNKTVFRSTNFAGYIGMVSGVKPDLFTLTLMNERFSLDGGYI 234
             * ::. *:   :*::: ::.* :: :  *.**:*: .*.**. *::: ::**   **:

Human       GILEWILGK-KDVMWIGFLTRTVLENSTSYEEAKNLLTKTKILAPAYFILGGNQSGEGCV 293
NMR         GLLEWILGK-KDASWIGFITRSVLENATSYEEAKNILAKTKLLAPAYFILGGNQSGEGCV 293
CMW         GVIEWILGK-KDAKWIGFIIRSVLENSTSYEEAKTILTKSKILAPAYFILGGSKSGEGCV 293
X.Laevis    GVLEWILGK-RDGMWMSFLTRSVLENATSYEEAKTLLSKTKLLAPAYFILGGNKSEEGCV 293
Mouse       GILEWMFGR-KDAQWVGFITRSVLENTTSYEEAKNTLTKTKIMAPVYFILGGKKSGEGCV 292
C.Elegans   GILKWVFGLEADGKWMSWLARETLETKTTYLDAKEHLMNTPMLSPVYFILGGSKKDEGCI 291
Bovine      GVMEWILGK-KDAQWVGFIIRSVLENSTSYEETKNILTKTKILAPAYFILGGNQSGEGCV 293
Cat         GIIEWILGK-KDAMWIGFITRSVLENSTSYEEAKNILTKTKILAPAYFILGGNKSGEGCV 293
Chicken     GIFEWFLGR-RDGMWMGFLTRSVLENATSYQDAKDKLAKTRLLAPAYFILGGKNSGEGCV 293
            *:::*.:*   *  *:.:: * .**. *:* ::*  * :: :::*.******.:.  ***:
```

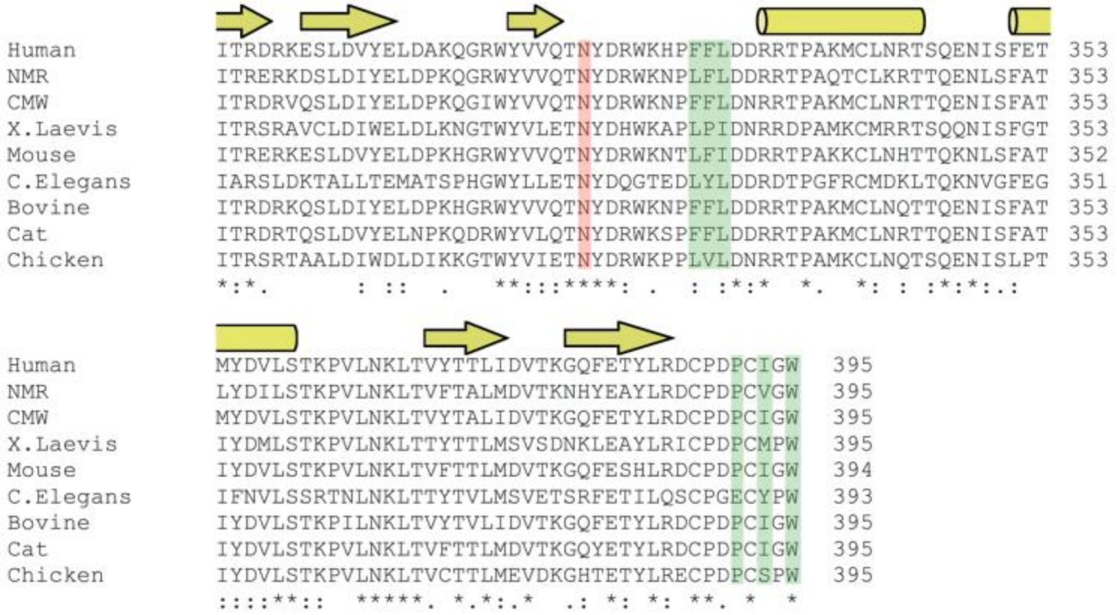

Supplementary Figure 4| Sequence alignment of aCDase orthologues. Structure based secondary structure is indicated at the top of the sequences. Residues highlighted in green are from the conserved hydrophobic surface surrounding the substrate binding site. Residues highlighted in red are from the enzyme active site.

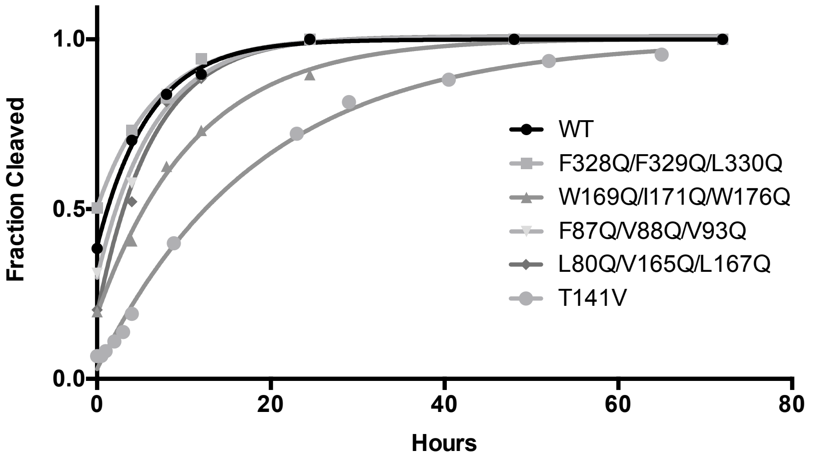

Supplementary Figure 5| Autocleavage of aCDase. Comparison of the autocleavage rate of wild-type (WT) aCDase with that of different mutants.

A

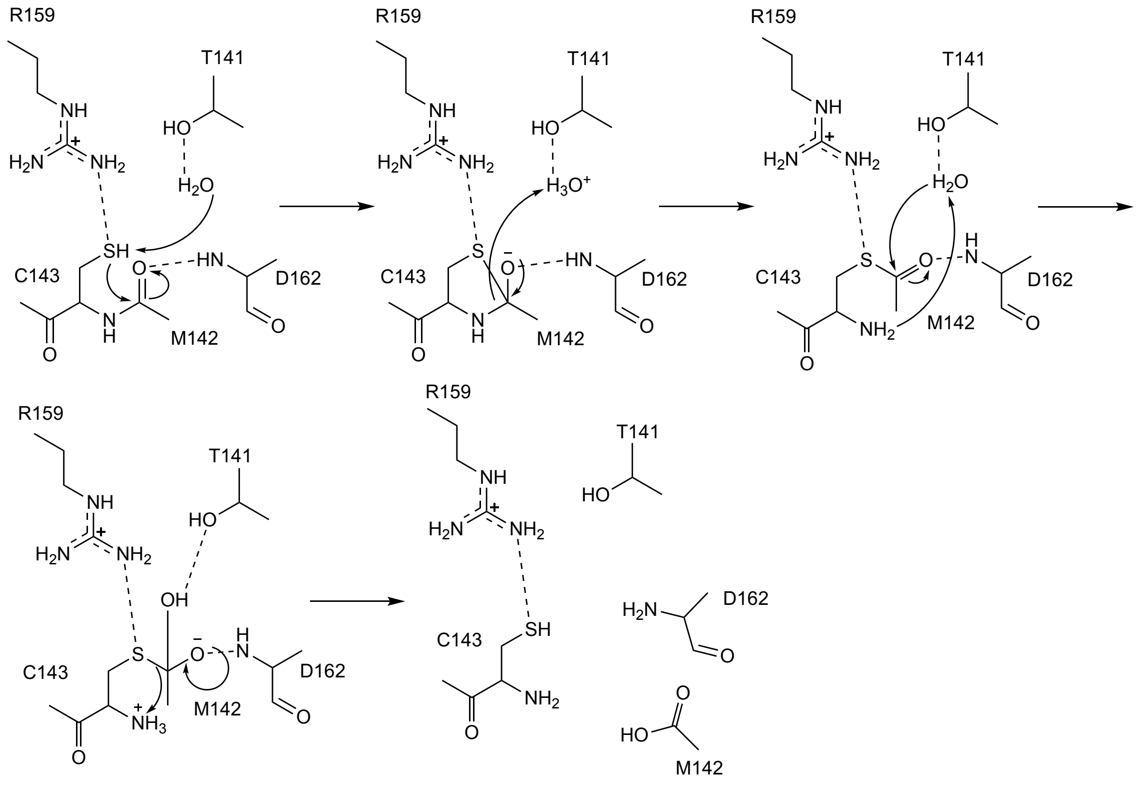
R159
T141
C143
M142
D162
H2O
H3O+
SH
NH2
NH3
HO
OH

OR

B

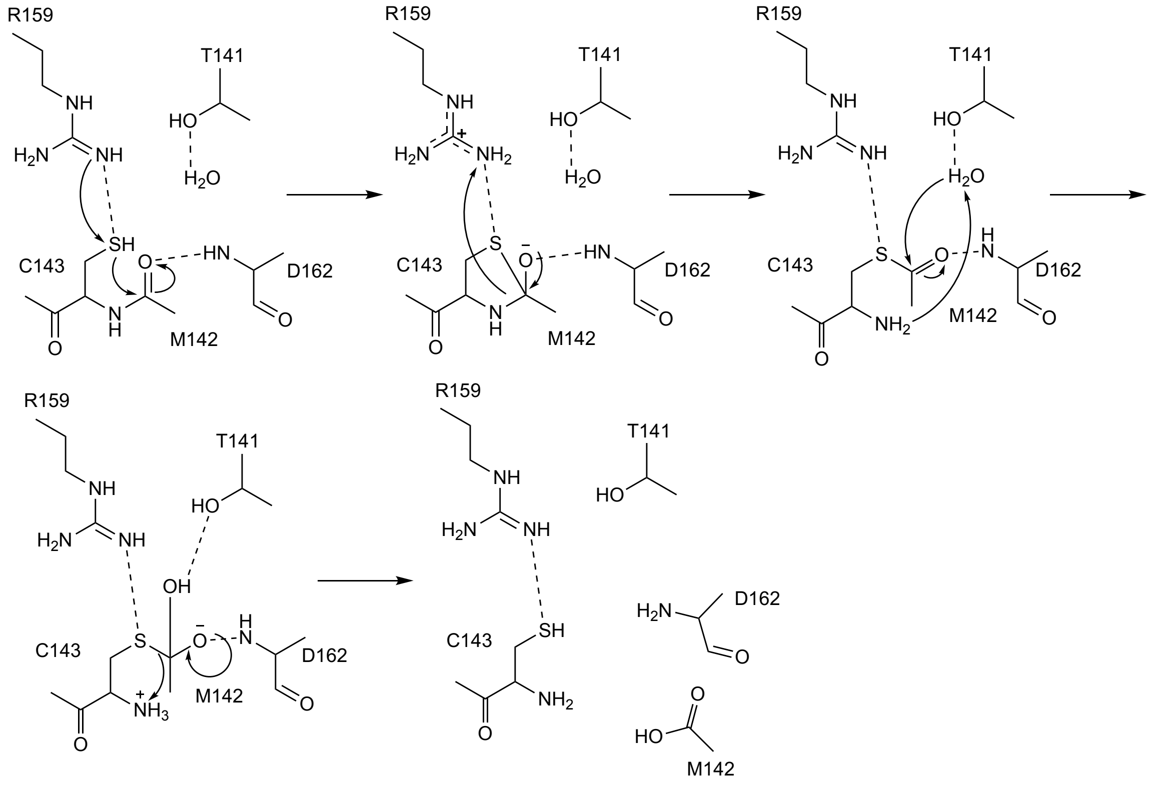
R159
T141
C143
M142
D162
H2O
SH
NH2
NH3
HO
OH

Supplementary Figure 6| Mechanism of aCDase autocleavage. Schematic of the proposed mechanism of autocleavage if (**A**) water or (**B**) Arg 159 act as the general base initiating the reaction.

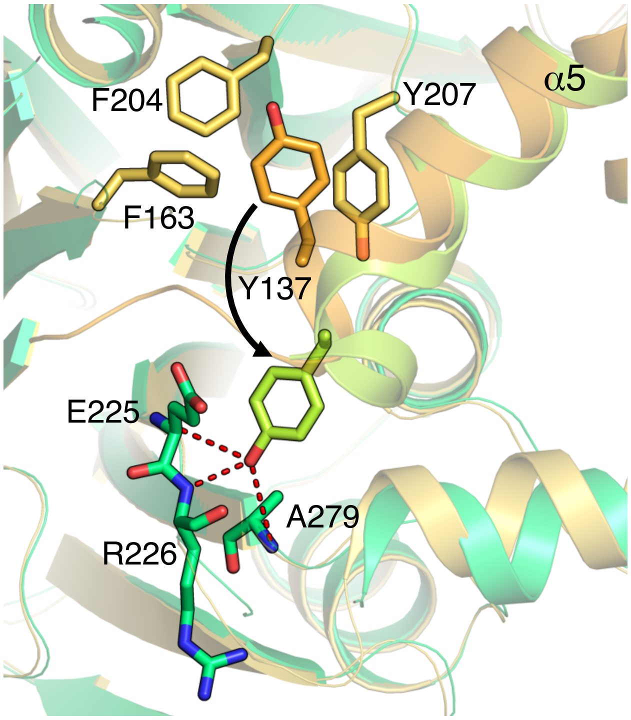

Supplementary Figure 7| Comparison of the microenvironment of Tyr 137 before and after autocleavage of aCDase. In the inactive form of the enzyme, Tyr 137 (orange sticks) is surrounded by hydrophobic residues (Phe 163, Phe 204, Tyr 207, Leu 211) from the β-subunit (yellow sticks). After cleavage, helix α5 is rotated by approximately 60°. In the mature aCDase, helix α5 bends upwards and Tyr 137 (light green sticks) is stabilized by hydrogen bonds between its hydroxyl group and the backbone atoms of three residues from the β-subunit (Glu 225, Arg 226, Ala 279).

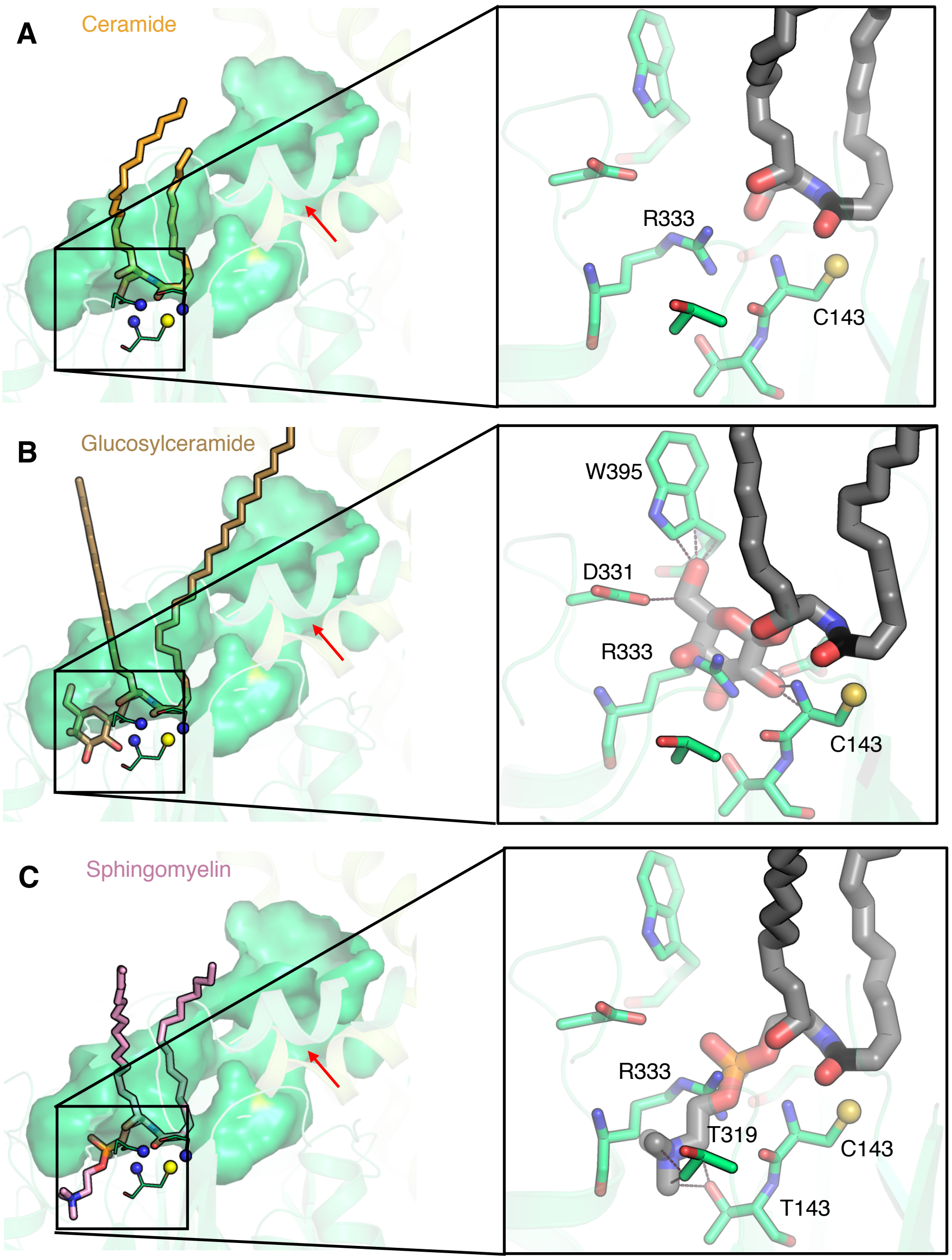

Supplementary Figure 8| Comparison between the docking of ceramide and other sphingolipids into the aCDase active site. (**A**) Ceramide (orange) fits snugly into the active site. Because of constraints imposed by the location of the active site cysteine (yellow sphere) and the oxyanion hole (blue spheres), the lipid head group faces away from the active site. These constraints prevent accommodation of the head groups of larger sphingolipids, such as (**B**) glucosylceramide and (**C**) sphingomyelin, into the cavity in a

manner similar to ceramide. The lateral cavity is labelled with a red arrow. In addition to Arg 333, clashes between protein residues and modeled lipids are indicated by red dashes in the enlarged panels.

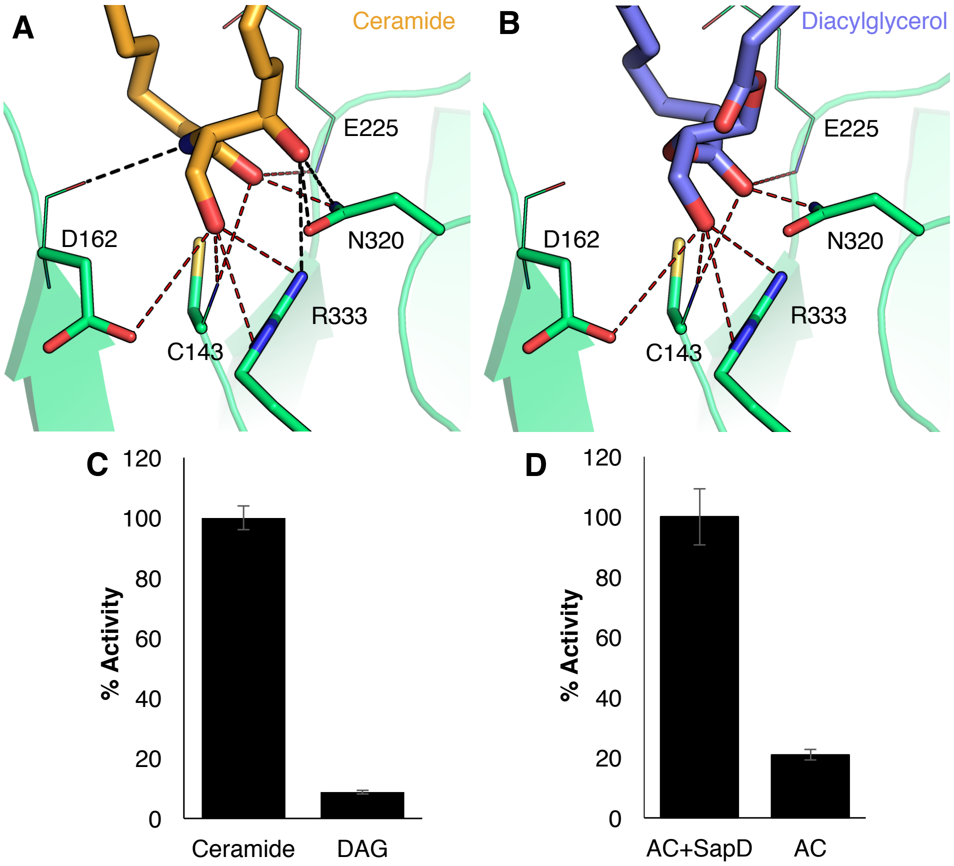

Supplementary Figure 9| Comparison of the interactions and activity of aCDase with ceramide and diacylglycerol (DAG), and activity of aCDase with or without Saposin D. (**A**) Docked ceramide (orange sticks) forms a total of 11 hydrogen bonds within the cavity of aCDase, whereas (**B**) manually docked DAG only forms 6 hydrogen bonds. Dashed lines represent hydrogen bonds, with ceramide-specific contacts in black. (**C**) ACDase activity on ceramide or DAG in Triton X-100 at pH 4 in the presence of the co-factor saposin D. 100% activity for wild-type enzyme corresponds to 0.54 μM ceramide hydrolyzed per nM protein per hour. Data are the means and standard deviations of six replicates. (**D**) Comparison of the activity of aCDase in the presence or absence of saposin D. Data for the ceramidase activity assay are the means and standard deviations of eight replicates.

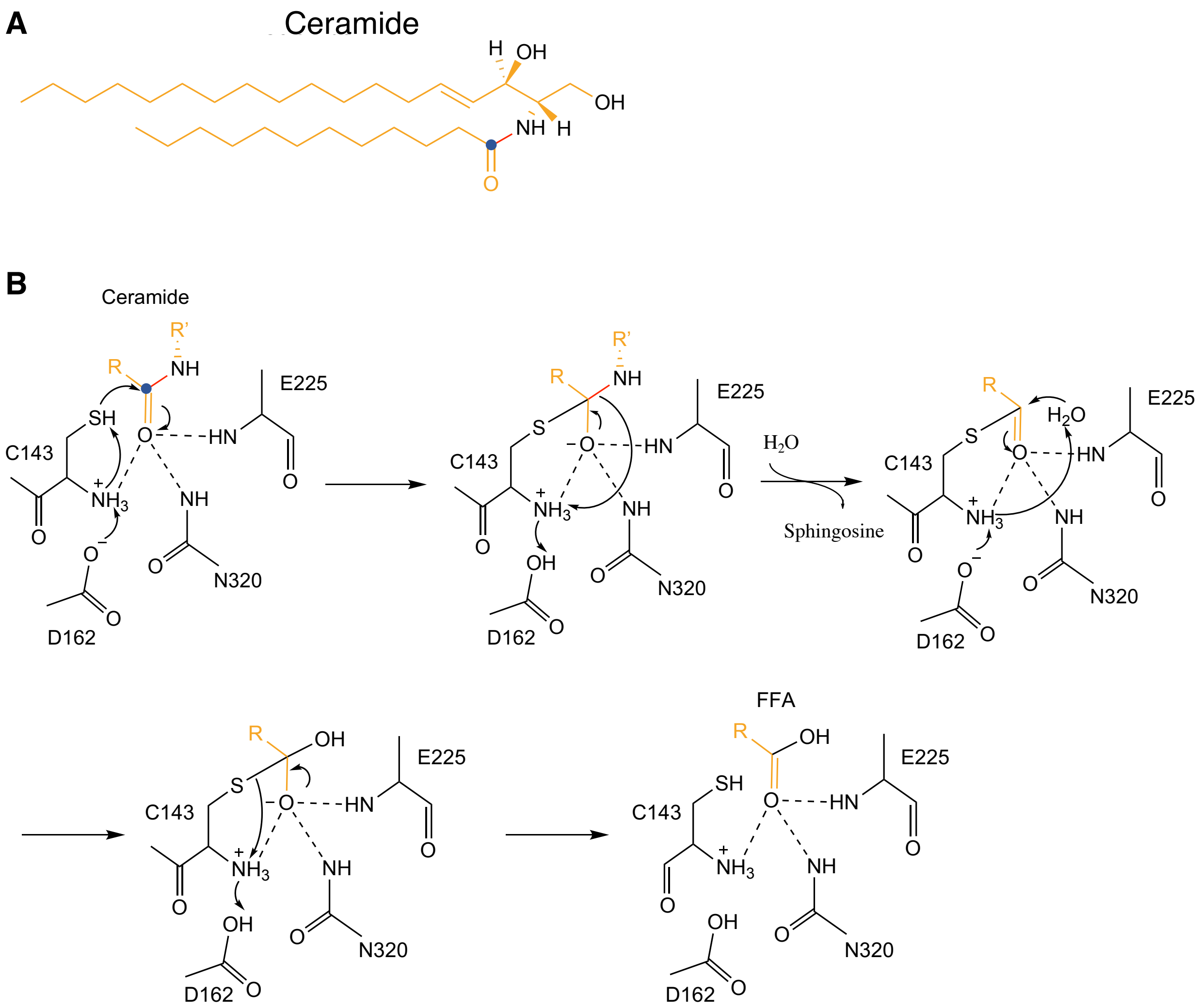

Supplementary Figure 10| Substrate hydrolysis mechanism of aCDase. (**A**) Chemical structure of the ceramide substrate of aCDase. (**B**) Proposed catalytic mechanism of aCDase ceramide hydrolysis. FFA, free fatty acid.

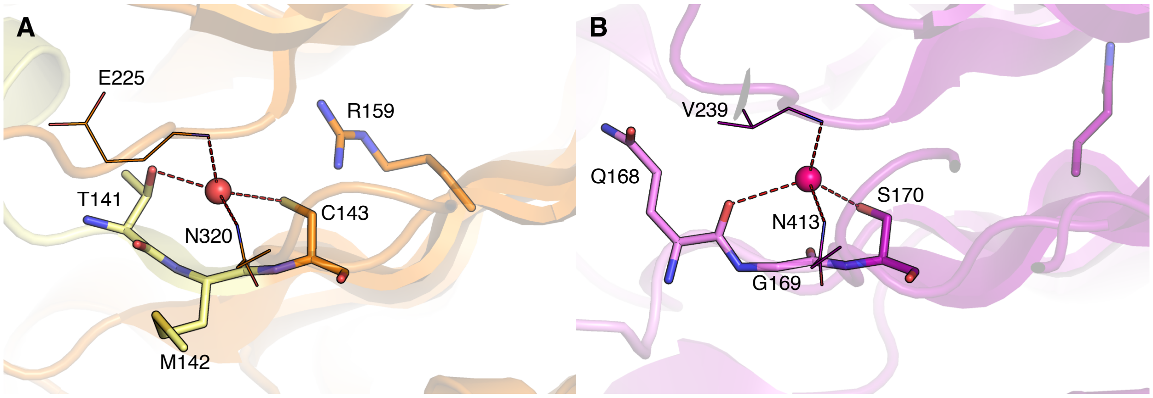

Supplementary Figure 11| Comparison of the active site-bound water molecule between aCDase and cephalosporin acylase (CA). (**A**) In the inactive form of aCDase, W1 (red sphere) forms four hydrogen bonds and is positioned adjacent to the catalytic Cys 143 for potential deprotonation and activation. (**B**) In CA (purple), the active site water molecule also forms four hydrogen bonds, placing it close to the catalytic Ser 170 for deprotonation and activation.

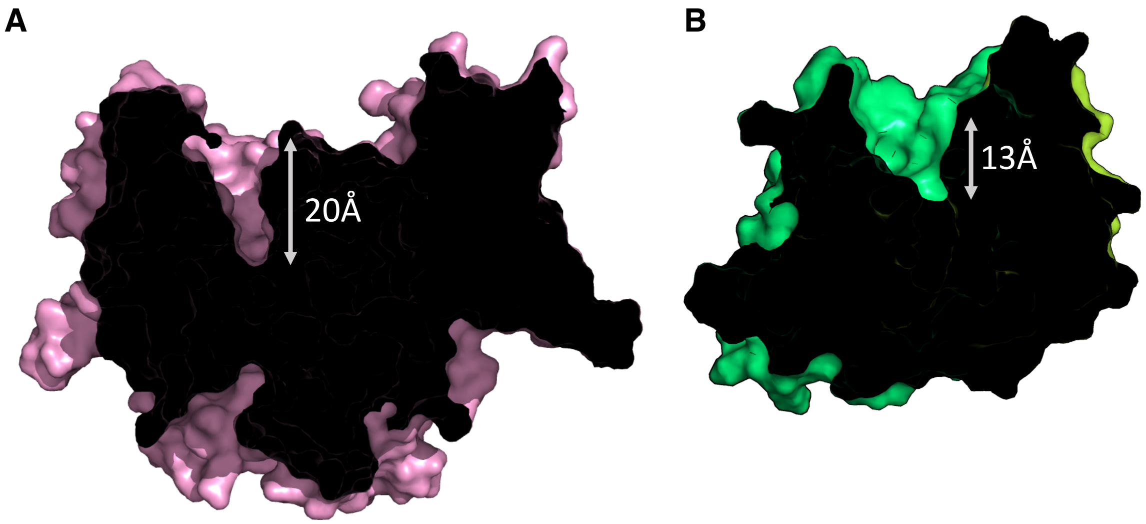

Supplementary Figure 12| Comparison of the active site cavity of the neutral and acid ceramidases. (**A**) nCDase has a 20Å-deep cavity leading to its active site, whereas (**B**) the activated aCDase has a 13Å-deep cavity, which could explain the difference in specificity in the optimal substrate length of these two enzymes (C12-ceramides for aCDase versus C16-ceramides for nCDase).

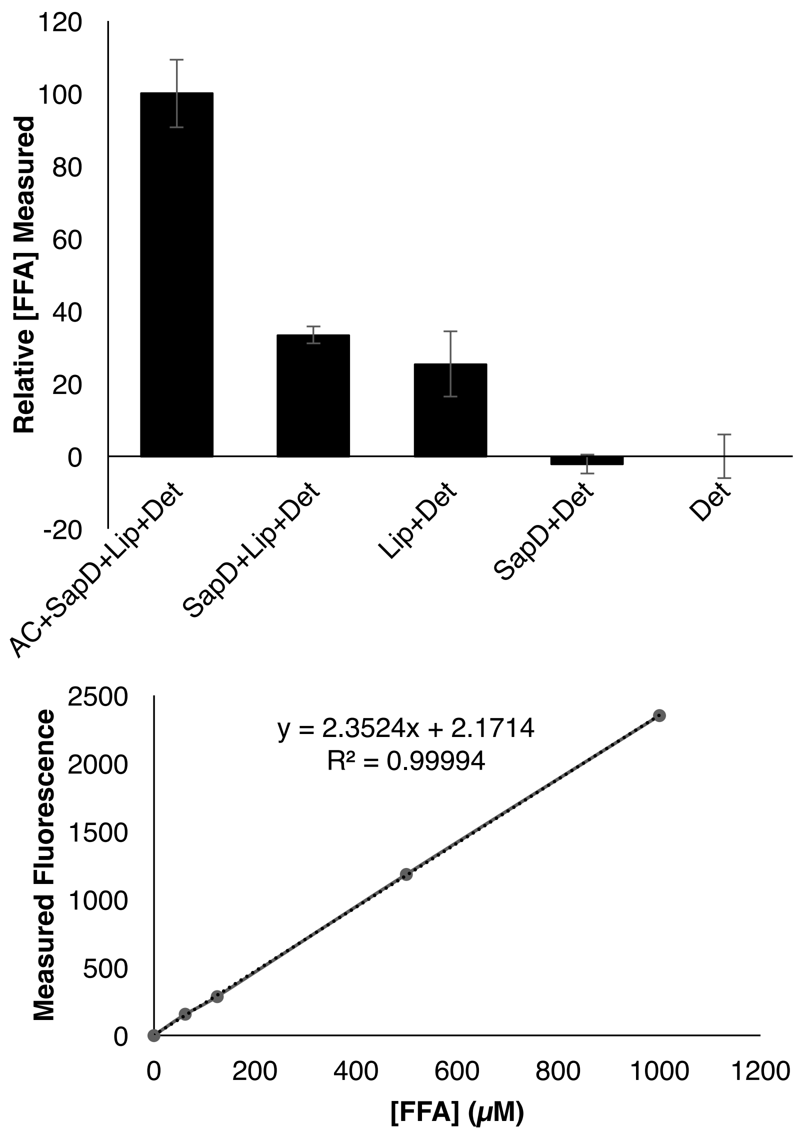

Supplementary Figure 13| Validation of the liposomal assay. The measured fluorescence was assessed to determine the contributions of the various components of the assay. AC=Acid Ceramidase; SapD=Saposin D; Lip=Liposomes; Det=Detergent (Triton X-100). The activities of the acid ceramidase in the presence of SapD presented in Figures 4B, 6C, and Supplementary Fig. 6B were corrected with the SapD+Lip+Der sample as background; the activity of acid ceramidase in the absence of SapD (Supplementary Fig. 6B) was corrected with the Lip+Det sample as background. Data for the validation assay are the means and standard deviations of six replicates.

Variants altering disulfide bond formation or glycosylation									
Probably Benign		N195S	S260N	N286S	N348S				
Probably Harmful		N342D	C392Y						
Variants altering the hydrophobic surface									
Probably Benign		L80V	M83L	M83T	I84M	V88L	I83V	M94I	M94T
L101F	F140C	I142V	V165A	F166L	I229V	Y233H	Y233F	L234M	I236V
F329V	L330F								
Uncertain		P88R	L237P	P391R	I393R				
Variants altering the surface									
Probably Benign		P26N	T28I	D30N	D30Y	G45D	A46T	P48S	P48S
T51A	T51I	H63Y	E64G	E64K	L67F	D68E	D68H	P71A	V72M
K74N	S79T	K81T	N82D	N85D	K100N	G103A	G103D	N107S	P109R
P109H	P109L	G110S	A121T	D124A	D124E	P126T	E129K	S132L	D150E
N170H	N174G	P184A	P184H	P184S	N194K	V198I	V198L	K200R	I240L
I240S	D245G	S262R	S262G	V304L	K310Q	Y321H	R323H	K325E	H326Y
P327R	T335M	P336A	P336H	P336S	L341V	R343C	T344I	S345R	S345N
S345G	E352K	Q380H	E382K	P389R	G394R				
Uncertain		P38L	D55E	P57R	P57L	D68G	Q95P	N170Y	D190A
L218P	S228G	D308Y	W314C	Y321D	R323C	R323L	R343P	T344P	D390G
Probably Harmful		Y36D	Y36H	R44S	R226H	W314R	D387Y		
Non-surface variants									
Probably Benign		I52V	I76V	D98E	D98H	F108L	I119M	I119V	I125K
I125T	I178L	T186A	T186S	F199L	A201G	Y207F	V208L	V208M	M210T

L211V	G213A	F214L	V246A	I249T	T253I	T253S	L257M	I282V	L283V
I294M	M354T	M354V	V369I	T372A	T374L				
Uncertain		T123N	N134S	V187M	Y207C	S220G	Y280C	G291V	L385P
Probably Harmful		G128R	Y137C	S145L	H157Y	N160D	K183E	G206D	T222R
R296Q	R296G	S359L	T360I	T368I	T371I	T371K	D375G	D375V	R386Q
R386W									

Supplementary Table 1| Predicted effects of aCDase variants.
